# Machine learning based approaches for structure activity relationship analysis of heparanase inhibitors

**DOI:** 10.1038/s41598-026-55098-4

**Published:** 2026-05-29

**Authors:** Rachana V. Shanbhogue, Neha S. Gandhi, Shanthi P. B., Sandhyalaxmi G. Navada

**Affiliations:** 1 Manipal Institute of Technology, Manipal Academy of Higher Education, 576104 Manipal, Karnataka India; 2 Department of Biotechnology and Chemical Engineering, Manipal University Jaipur, 303007 Rajasthan, India; 3 School of Biomedical Sciences, Queensland University of Technology, Kelvin Grove, Queensland Brisbane, Australia

**Keywords:** Cancer, Computational biology and bioinformatics, Drug discovery

## Abstract

Human Heparanase (HPSE), the only mammalian endo-$\beta$-D-glucuronidase, plays an important role in extracellular matrix remodeling and the release of heparin-bound growth factors. Its overexpression is strongly correlated with increased tumor growth, angiogenesis, metastasis, and inflammation, highlighting HPSE as a compelling therapeutic target for oncology and inflammatory diseases. This study aimed to develop and validate a robust computational workflow for predicting the activity class of potential HPSE inhibitors using curated data from the ChEMBL database. Bioactivity data ($pIC_{50}$) for known HPSE inhibitors were extracted and put through a meticulous data curation process, which included chemical structure standardization, molecular weight filtering, and final deduplication based on standardized isomeric SMILES to ensure structural uniqueness. Continuous $IC_{50}$ values (nM) were converted to $pIC_{50}$ and subsequently categorized into three activity classes: A ($pIC_{50} \ge 4$ and $< 5$), B ($pIC_{50} \ge 5$ and $< 6$), and C ($pIC_{50} \ge 6$ and $< 7$) for multi-class classification. Molecular representations included two-dimensional physicochemical descriptors, Morgan fingerprints, and three-dimensional descriptors derived from optimized low-energy conformers generated using ETKDGv3 and MMFF94s. Multiple machine learning classifiers were evaluated using pipelines incorporating imputation, scaling, optional Principal Component Analysis (PCA) dimensionality reduction applied to the combined feature sets, and SMOTE (Synthetic Minority Over-sampling Technique) to address class imbalance. Models were trained and optimized using randomized search cross-validation on an 80% training split, maximizing balanced accuracy. The best-performing model pipeline (RF_B, a Random Forest with PCA on 2D+Morgan Fingerprints+3D features) achieved approximately 80% accuracy and 78.5% balanced accuracy on the held-out 20% test set. The final validated model was successfully utilized to predict the activity classes of new, unseen compounds. This comprehensive pipeline provides a validated tool for classifying HPSE inhibitors derived from ChEMBL data, potentially aiding virtual screening efforts and guiding hit prioritization in drug discovery campaigns targeting HPSE.

## Introduction

Heparanase (HPSE) has emerged as a promising therapeutic target since it plays a significant role in regulating a variety of pathological processes such as extracellular matrix breakdown, growth factor release, and regulation of inflammation^1,2,37,40^. Its overexpression is strongly associated with cancer growth, metastasis, and chronic inflammation as outlined in research regarding sarcoma and other cancers^3,4,34,35,36,50^. The central role of the enzyme in promoting disease development, further complicated by regulatory networks including its homologue Hpa2^5^, renders its inhibition an attractive strategy, as it may interrupt several pathological pathways simultaneously. This broader potential has enabled sustained activity toward HPSE inhibitor identification and development.

Considerable effort has been directed toward HPSE inhibitor discovery^6,45,49^, resulting in multifaceted strategies. Initial approaches involved structural analogs to the native substrate, heparan sulfate (HS), resulting in chemically modified heparin fragments (e.g., Roneparstat, Muparfostat), sulfated synthetic compounds (e.g., PG545), and neutralizing antibodies^3,4,7^. Although effective in preclinical models, these large, structural molecules are frequently plagued by poor pharmacokinetics and unwanted off-target effects through binding to other heparin-binding proteins, inducing side effects such as anticoagulation^1,7^. As a result, the identification of strong, selective, and orally bioavailable small-molecule inhibitors is a fundamental goal, although this means facing specific structure-based design problems^8^.

In response to these challenges, computational methods have revolutionized drug discovery, offering robust means for the prediction of biological activity and the effective searching of huge chemical spaces^9–11,41,42,43,44,54,46,55,57^. Although classical Quantitative Structure-Activity Relationship (QSAR) modeling is a useful paradigm, machine learning (ML) methods are well-suited to deal with the high-dimensional and often non-linear patterns present in structure-activity data^10^. This has been convincingly shown by the successful prediction of models for inhibitors of other prominent enzyme targets, including aromatase, LSD1, acetylcholinesterase (AChE), and $\alpha$-glucosidase, frequently by employing ensemble methods such as Random Forest and XGBoost^12–17^.

The rationale for using ensemble-based classifiers such as Random Forest (RF) and Extra Trees (ET) is further supported by their proven efficacy in broader bioinformatics applications. For instance, the SOLVE (Soft-Voting Optimized Learning for Versatile Enzymes) predictor demonstrates that ensemble frameworks, which integrate RF with other decision-based models, can successfully achieve high-throughput enzyme function annotation and identify functional motifs via interpretability tools like SHAP^18^. While emerging architectures such as GTransCYPs utilize Graph Neural Networks (GNNs) combined with transformer mechanisms to predict CYP450 enzyme inhibitors^19^, such models often require substantial datasets to fully leverage native graph processing. In contrast, ensemble methods remain benchmarks for enzyme-inhibition studies due to their superior data efficiency and structural interpretability when operating on the specialized, relatively limited chemical spaces typical of HPSE inhibitors.

The predictive power of any machine learning model is intrinsically linked to the quality and richness of its molecular representations. While traditional 2D physicochemical descriptors offer a source of information on properties such as solubility and size, the field has rapidly moved toward employing techniques that are far more sophisticated. The use of topological fingerprints, including Morgan fingerprints (MF), in combination with new features derived from areas such as persistent homology or ’hybrid’ similarity measures, becomes imperative in enhancing predictive performance^20–23,33,51,47,52,58^. Besides, the use of 3D descriptors that capture critical features of molecular shape, volume, and conformer stability should become of paramount importance when performing drug discovery against targets like HPSE, whose interaction is highly complex within a three-dimensional enzyme pocket. To this end, a set of varied and comprehensive features across 2D, 3D, and abstract fingerprint space will guarantee that the model is able to holistically capture the complex structural requirements for potent inhibition of HPSE^23^.

Constructing a robust and stable ML model involves a well-thought-out workflow.[ ^38^ ] An important step is solving the issue of imbalanced datasets, which is prevalent in biological data, using methods such as the Synthetic Minority Over-sampling Technique (SMOTE) being an industry-standard method^24,56^. Aside from data processing, model prediction reliability is of top importance. This requires open validation protocols that guarantee model replicability and, most importantly, the determination of the model’s Applicability Domain (AD). A clear definition of AD delineates the chemical space where a model’s predictions can be relied upon, an essential step toward avoiding overly optimistic outcomes and enabling operational deployment of models in virtual screening campaigns^25–27^.

The ultimate goal of drug discovery pipelines considering computational methods is not only to yield high accuracy scores but also to achieve translational utility-meaning the models have to identify drug candidates reliably that can proceed to expensive preclinical testing in the real world. The only way a model will be truly useful for predicting Heparanase (HPSE) inhibitors is by moving beyond a good performance on familiar training data.

This requires two major methodological safeguards, namely, external validation and definition of the Applicability Domain. Only strict external validation will enable realistic modeling performance estimation using new and structurally different chemical sets. Of equal importance is a clear definition of the AD, representing the boundaries of chemical space in which a model’s predictions are reliable. When predictions extend to molecules outside this defined domain, they can be completely unreliable, with significant time and resources wasted in virtual screening campaigns. Thus, developing a useful, high-quality HPSE model needs to be built on transparent protocols that confirm its robustness, generalizability, and chemical relevance across diverse compound structures.

**Fig. 1:**
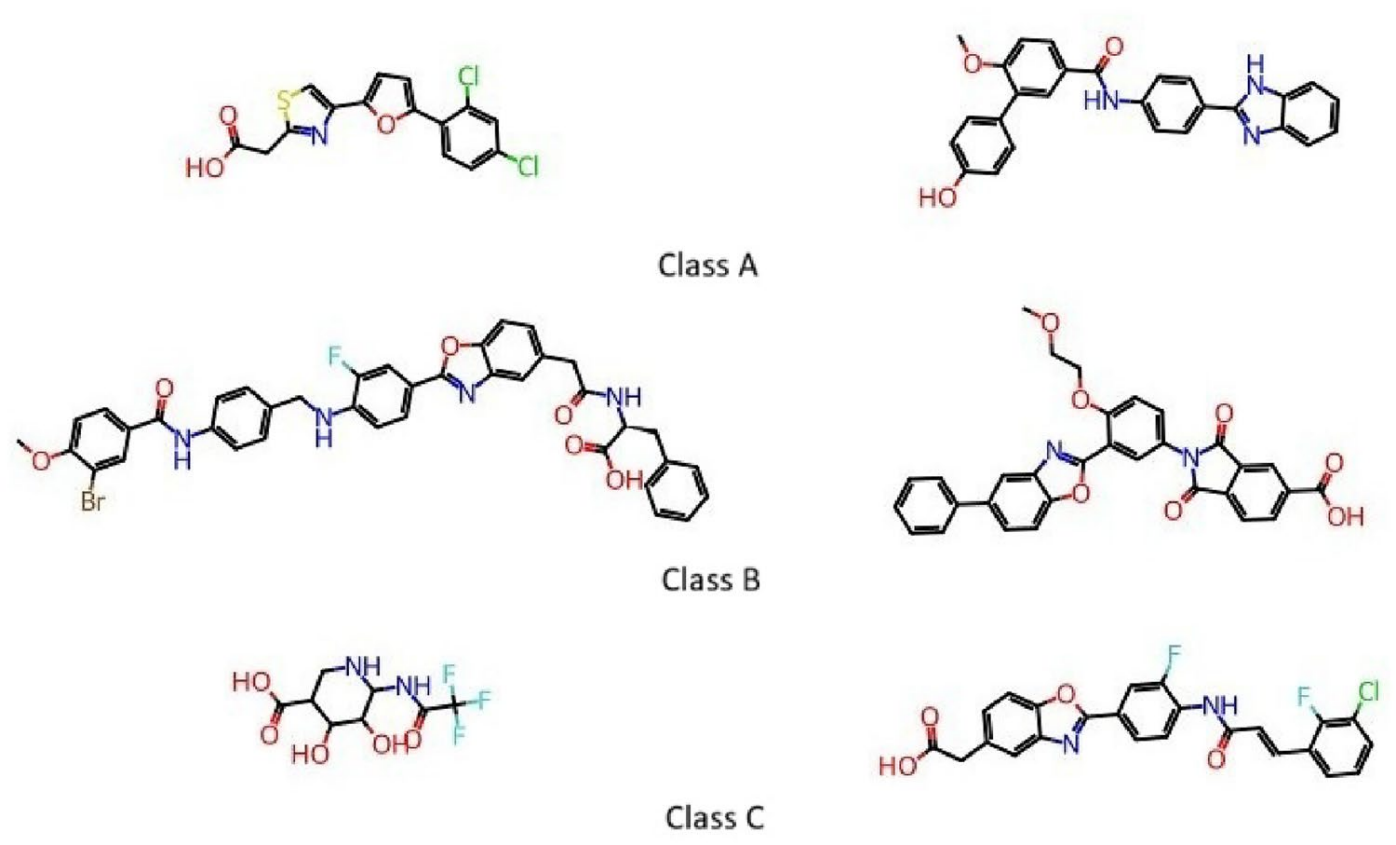
The figure displays example molecules belonging to Class A ($pIC_{50}$ [4–5)), Class B ($pIC_{50}$ [5–6)), and Class C ($pIC_{50}$ [6–7)) from the final modeling dataset, providing an overview of the types of chemical structures associated with different potency ranges.

In spite of the recognized therapeutic potential of HPSE and general advances in applying ML for enzyme inhibitor discovery, two underpinning gaps in the literature persist. First, there is a considerable absence of robustly validated, openly accessible multi-class classification models that can categorize HPSE inhibitors into different potency classes. Second, thorough reports describing end-to-end ML pipelines for HPSE featuring careful stereochemistry-conscious data curation, rich feature engineering, and clear comparison of several classifiers are scarce. This research aims to address these gaps by establishing and evaluating such a pipeline, delivering a reliable predictive model for HPSE inhibitor class prediction and prioritization, with representative molecules for each class shown in Fig. 1.

## Methods

### Data collection

Biological activity data pertaining to inhibitors of HPSE (UniProt: Q9Y251, ChEMBL: CHEMBL2719) were collected from the ChEMBL database (Version 35, accessed 19/05/2025)^28^. The study centered on $IC_{50}$ (half-maximal inhibitory concentration) bioactivity measurements reported in nanomolar (nM) and records for which the standard relation indicated an exact value. For compounds with multiple $IC_{50}$ values, the entry closest to the compound’s median $IC_{50}$ was retained. Structures, represented as canonical SMILES, were processed using RDKit (v. 2024.09.2)^29^, involving standardization (cleanup, largest fragment, charge neutralization), a molecular weight (MW) filter (MW $\le$ 500 Da), and rigorous stereochemistry-preserving standardization yielding isomeric SMILES. The dataset was then deduplicated based on these unique isomeric SMILES. Oligosaccharide-based molecules such as PG545 and their derivatives were not considered in this study. All $N=228$ unique standardized SMILES were successfully processed by RDKit; however, three compounds were excluded from the final modeling dataset because they fell into the ’Class D’ ($pIC_{50} \ge 7.0$) outlier category. This exclusion ensured that the model focused on the primary activity spectrum ($4.0 \le pIC_{50} < 7.0$) without being skewed by extreme potencies.

Inhibitory potency was expressed as $pIC_{50}$, calculated from $IC_{50}$ values (nM) using Equation (1):

1 $$\begin{aligned} pIC_{50} = -\log _{10}(IC_{50, \text {M}}) \end{aligned}$$

where $IC_{50, M}$ is the molar concentration ($IC_{50, nM}$ $\times$ 10$^{-9}$).

For subsequent multi-class classification, compounds were categorized based on their $pIC_{50}$ values into: Class A (4.0 $\le$ $pIC_{50}$ < 5.0), Class B (5.0 $\le$ $pIC_{50}$ < 6.0), and Class C (6.0 $\le$ $pIC_{50}$ < 7.0).These thresholds were selected based on established medicinal chemistry conventions for potency tiers in early-stage drug discovery. A $pIC_{50}$ value of 6.0, corresponding to an $IC_{50}$ of $1\mu M$, is a widely accepted boundary for ’hit-to-lead’ potency, distinguishing high-potency candidates from moderate hits. Similarly, a $pIC_{50}$ of 5.0 ($10\mu M$) serves as a standard lower-bound cutoff for defining biologically relevant activity in primary high-throughput screening. By utilizing these logarithmic integers, the model effectively bins compounds into actionable pharmacological categories: High Potency ($< 1\mu M$), Moderate Potency ($1-10 \mu M$), and Low Potency ($> 10\mu M$). Compounds falling outside these defined ranges, specifically those with $pIC_{50} \ge 7.0$ (Class D) or $pIC_{50} < 4.0$ (’Other’), were excluded to preserve clear, distinct activity boundaries for the three primary classes. We chose to exclude these extreme values to mitigate the impact of activity cliffs and focus the model on the most experimentally relevant pharmacological range ($4.0 \le pIC_{50} < 7.0$). Compounds with $pIC_{50} < 4.0$ were considered inactive noise that could dilute the structural signal of the ’Low Potency’ class, while super-potent outliers ($\ge 7.0$) were removed to prevent the model from being skewed by extreme specificities rather than generalizable structural rules. However, this exclusion may limit the model’s ability to identify highly potent inhibitors, which are often of interest in later-stage drug discovery. This resulted in a final modeling dataset of N=225 inhibitors, with the distribution: Class A (n=18), Class B (n=116), and Class C (n=91). These categorical labels were numerically encoded (0, 1, 2) for machine learning using Scikit-learn (v. 1.5.1). This distribution highlights a significant natural class imbalance, particularly for the minority Class A, which was specifically addressed during model development to ensure predictive reliability across all potency levels.

### Molecular descriptor and fingerprint generation

For numerically encoding the chemical structures of the N=228 hand-curated compounds into machine learning, a representative collection of molecular descriptors and fingerprints was computed by using the RDKit library.

The standardized SMILES strings were cleaned by turning them into molecule objects and then processing them to fix common issues like removing extra parts and confirming the molecules are neutral. Any molecules that couldn’t be properly processed were removed to keep the data reliable.

Three broad feature categories were then calculated:

#### 2D Descriptors:

208 standard descriptors for capturing physicochemical descriptors (e.g., Topological Polar Surface Area (TPSA), BalabanJ, RingCount), electronic descriptors, and topological indices describing molecular connectivity and branching.

#### Morgan Fingerprints:

These spherical fingerprints, like ECFP4, were developed to describe certain substructural features. The atomic environments within 2 bonds were counted and hashed to a fixed-length 2048-bit vector, giving a rich representation of local chemical environments that also implicitly contain stereochemical information.

#### 3D Descriptors:

From the energy-minimized 3D conformers, descriptors accounting for molecular shape, size, and symmetry were computed.For each molecule, initial 3D conformers were generated using the ETKDGv3 algorithm within RDKit (v2024.09.2) with a fixed randomSeed=42 and chirality enforced. These conformers were then energy-minimized using the MMFF94s force field with a maximum of 200 iterations to reach a local energy minimum. From these optimized 3D conformers, descriptors accounting for molecular shape, size, and symmetry were computed, including parameters such as Asphericity, Eccentricity, Inertial Shape Factor, and Principal Moments of Inertia.

Final feature vectors containing the merged 2D descriptors, 2048-bit Morgan fingerprints, and 3D descriptors (2268 informative features in total after initial processing) were aggregated with compound identifiers and activity data into a merged dataset ready for subsequent machine learning and exploratory analysis.

### Clustering analysis and feature space visualization

To investigate the chemical space coverage and ensure dataset diversity, a two-stage clustering process was performed. We utilized 1024-bit Morgan fingerprints for clustering (*t*-SNE/DBSCAN) to reduce feature sparsity, which improves the visual density and separation of clusters in the $2\text {D}$ projection. This clustering analysis served a well-defined purpose: to confirm that the HPSE inhibitors were structurally diverse and that the subsequent stratified sampling for model training effectively covered all structural families in the dataset. In contrast, 2048-bit fingerprints were used for machine learning modeling (as described in the following section) to maximize structural resolution and minimize bit collisions, ensuring the classifiers operated with the highest possible information density.Clustering and dimensionality reduction were performed using the Scikit-learn (*v*1.5.1) library in Python. Clustering and dimensionality reduction were performed using the Scikit-learn (v1.5.1) library in Python^30^. First, *t*-Distributed Stochastic Neighbor Embedding (*t*-SNE)^31^ mapped the compounds to two dimensions according to their 1024-bit Morgan fingerprints (radius 2) generated via RDKit (v2024.09.2)^29^. The *t*-SNE parameter options were $\text {perplexity} = 20$, ’pca’ initialization, ${\mathrm{random}}\_{\text{state = 42}}$, and 1000 iterations^31^. Subsequently, Density-Based Spatial Clustering of Applications with Noise (DBSCAN)^32^ was applied to these 2D *t*-SNE coordinates using an epsilon ($\epsilon$) of 1.25 and ${\mathrm{min}}\_{\mathrm{samples}}$ of 3, identifying 29 distinct clusters and 29 noise points. These cluster assignments were used to color the t-SNE plot for visualization. For each identified actual cluster, characteristics including size, intra-cluster Tanimoto similarity (based on 2048-bit Morgan fingerprints, radius 2), and Maximum Common Substructure (MCS) were calculated, and representative molecular structures were visualized to aid interpretation.

### Machine learning model development

Predictive classification algorithms were built from the final curated and featurized dataset N=225 HPSE inhibitors, which belonged to activity Classes A (n=18), B (n=116), and C (n=91). This dataset was split into a training set (80%, n=180) and an independent test set (20%, n=45) through stratified sampling (random_state=42) to preserve class distribution.

Automated modeling pipelines were created using the imbalanced-learn library to provide stable preprocessing and classifier training. Both pipelines involved sequential operations: (1) median imputation of missing feature values; (2) removal of low-variance features (variance threshold = 0.01); (3) standardization of features to zero mean and unit variance by StandardScaler; and (4) use of the Synthetic Minority Over-sampling Technique (SMOTE, k_neighbors=5, random_state=42) for class imbalance, used dynamically within only training cross-validation folds.Due to the natural class imbalance (Class A $n=18$, Class B $n=116$, Class C $n=91$), we implemented SMOTE within an ImbPipeline. This approach ensures that the decision boundaries of the Random Forest and XGBoost models are not overwhelmed by the majority classes, allowing for more accurate classification across the entire activity spectrum. Pipeline A appended these directly to the classifier. Pipeline B added a further Principal Component Analysis (PCA) step after standardization but prior to SMOTE, preserving components that explained at least 95% of the variance (random_state=42).

A variety of Scikit-learn classification models were tested: Logistic Regression, K-Nearest Neighbors, Support Vector Machine, Random Forest, Extra Trees (all set with class_weight=’balanced’ where appropriate), and Gradient Boosting (early stopping included). Hyperparameters for each algorithm-pipeline pair were tuned using RandomizedSearchCV on the n=180 training set alone. It ran 40 parameter sets (n_iter=40) per model, tested through 5-fold stratified cross-validation (shuffle=True, random_state=42), with optimization based on balanced_accuracy. Optimal hyperparameters of each configuration were determined, and the respective pipeline was retrained on the entire training set.

The performance of these optimized pipelines on generalization was then scrutinized on the hold-out test set of 20% (n=45). Standard multi-class classification metrics such as overall accuracy, balanced accuracy, macro-averaged precision, recall and F1-score were computed. Confusion matrices and classification reports were also created to give a fine-grained analysis of per-class performance on unseen data.

### Virtual screening

To demonstrate prospective applications, we began screening public databases available through the CHEESE DeepMedChem server (https://cheese.deepmedchem.com/), which together contain over a million compounds. The initial library of over 1 million compounds was filtered using the CHEESE database server, employing the potent heparanase inhibitor Roneparstat as the query structure for 3D electrostatic and shape similarity. Compounds were selected based on their Active Pair similarity, which represents a scoring metric derived from the pharmacophoric overlap and structural alignment between the query and the library molecules. Following this similarity-based prioritization, 900 compounds were retained for subsequent machine learning evaluation, While this similarity-based filtering enriches for HPSE-relevant compounds, it may introduce bias toward known scaffolds and limit the discovery of structurally novel inhibitors. Library compounds underwent identical standardization as the training set. Subsequently, 2D descriptors and 2048-bit Morgan fingerprints (radius 2) were calculated as their sole feature representation for this screening campaign. These feature vectors were then processed by the pre-fitted RF_B pipeline. Although the RF_B pipeline was originally trained on a broader feature set that included 3D descriptors, its internal preprocessing steps (including imputation, scaling, and PCA transformation learned from the full training feature space) allowed it to process the input derived from 2D and fingerprint features of the library compounds. The pipeline ultimately generated predicted probabilities for each library compound belonging to activity Classes A, B, or C. A virtual screening score, defined as the maximum predicted probability among these classes, was used to rank compounds. The recovery of two known active HPSE inhibitors included in the library was monitored.

## Results

### Characterization of the dataset

**Fig. 2:**
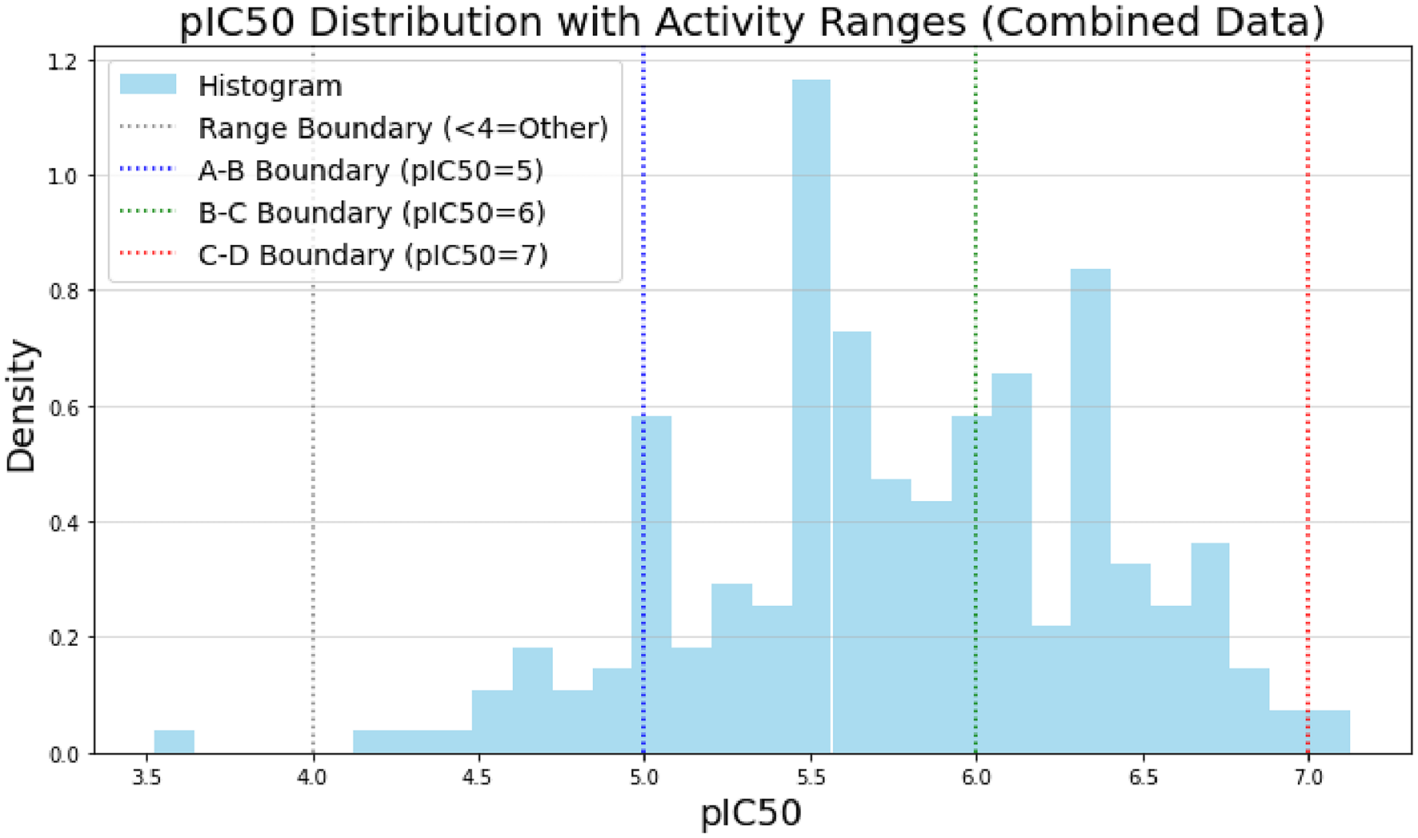
The distribution of $pIC_{50}$ values for the initial curated dataset of N = 228 unique HPSE inhibitors obtained from ChEMBL is shown in the histogram. The plot displays the frequency density across the $pIC_{50}$ range. Vertical dotted lines indicate the thresholds used to define activity classes for subsequent classification modeling: $pIC_{50}$ < 4.0 (“Other”, excluded from modeling), 4.0 $\le$ $pIC_{50}$ < 5.0 (Class A), 5.0 $\le$ $pIC_{50}$ < 6.0 (Class B), 6.0 $\le$ $pIC_{50}$ < 7.0 (Class C), and $pIC_{50}$ $\ge$ 7.0 (Class D excluded from modeling). This classification scheme allows for the differentiation of compounds based on potency, with Classes A through C included in the modeling process and Classes “Other” and D excluded due to their extreme low or high activity. The histogram illustrates the distribution of compounds relative to these defined potency thresholds.

The filtered dataset from ChEMBL, which was employed in all analyses that followed, contained N=225 unique human Heparanase (HPSE) inhibitors. The drugs showed a substantial range of inhibitory activity, having $pIC_{50}$ values across the specified activity classes, a feature fundamental to training efficient quantitative structure-activity relationship models. For ranking, the inhibitors were divided into: Class A ($pIC_{50}$ [4–5), n=18 inhibitors; Class B ($pIC_{50}$ [5–6), n=116 inhibitors; and Class C ($pIC_{50}$ [6–7), n=91 inhibitors. The $pIC_{50}$ value distribution, as shown in Fig. 2, is seen to clearly show this distribution, with the dominance of Class B and C inhibitors and the relative sparsity of Class A compounds. This natural class imbalance constitutes a recognized problem for machine learning algorithms, which require handling during model development.

**Fig. 3:**
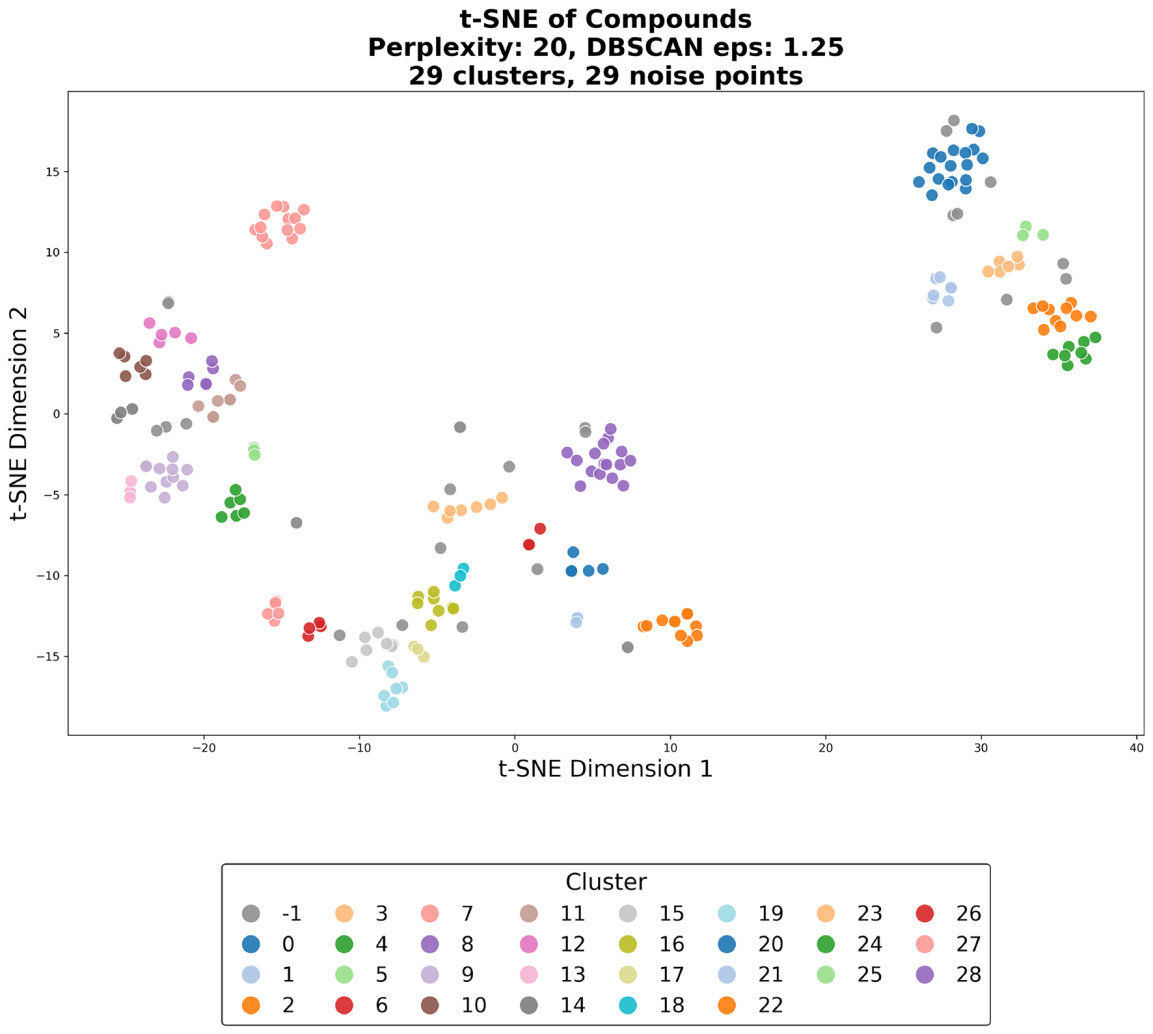
To map the association between structural features and HPSE inhibition in the N=225 compound set, t-Distributed Stochastic Neighbor Embedding (t-SNE) was used. It is a non-linear dimension reduction method that projects high-dimensional feature vectors into a two-dimensional space, maintaining local similarities and uncovering clustering patterns. t-SNE analysis was conducted on Morgan fingerprints (radius 2, 1024 bits) with a perplexity of 20. Further DBSCAN clustering (eps=1.25, min_samples=3) on the t-SNE embeddings detected 29 different structural clusters and labeled 29 compounds as noise points. The two-dimensional map from this is presented in Fig. 4, where close points indicate structural similarity according to the fingerprint description.

To map the association between structural features and HPSE inhibition in the N=225 compound set, t-Distributed Stochastic Neighbor Embedding (t-SNE) was used. It is a non-linear dimension reduction method that projects high-dimensional feature vectors into a two-dimensional space, maintaining local similarities and uncovering clustering patterns. t-SNE analysis was conducted on Morgan fingerprints (radius 2, 1024 bits) with a perplexity of 20. Further Density-Based Spatial Clustering of Applications with Noise (DBSCAN) clustering (eps=1.25, min_samples=3) on the t-SNE embeddings detected 29 different structural clusters and labeled 29 compounds as noise points. The two-dimensional map from this is presented in Fig. 3, where close points indicate structural similarity according to the fingerprint description.

**Fig. 4:**
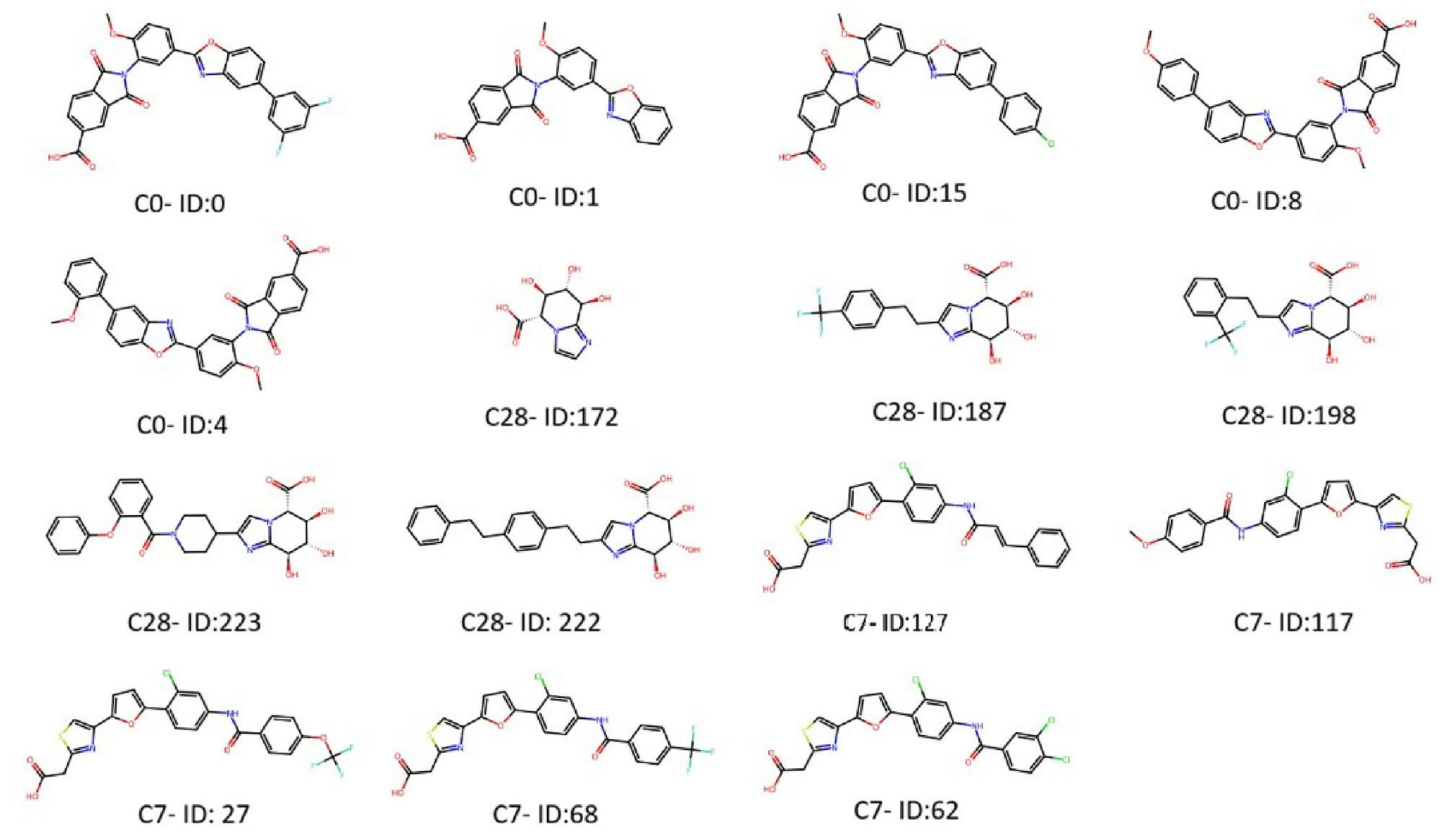
Representative 2D chemical structures from a few of the 29 DBSCAN-identified clusters (see Fig. 3, clusters C0-C28). The selection illustrates the structural diversity within the N=225 HPSE inhibitor dataset.

The t-SNE mapping (Fig. 3) shows various groupings of varying density and separation throughout the map. A few groups look like dense, solid islands, whereas others are diffuse or spread across multiple areas of the embedding. Noise points (gray) are spread around, indicating molecules that failed to strongly group with any large cluster at the given DBSCAN parameters. This representation implies significant chemotype-oriented heterogeneity in the dataset. The structural diversity within these 29 DBSCAN clusters is qualitatively depicted in Fig. 4, displaying representative 2D chemical structures for each group. Visual inspection of Fig. 4 verifies the significant chemical diversity within the dataset. For example, compounds that contain long aliphatic chains are dominant in some clusters (e.g., seen in those marked C20, C21, C22 in the sample image), whereas molecules that are defined by intricate, multi-ring aromatic or heterocyclic systems are dominant in others (e.g., dominant in clusters C0, C1, C2, C3). A few clusters, like C10 and C11, exhibit high visual intra-cluster homogeneity according to their representatives, indicating the existence of clearly defined structural families having a common core scaffold. This clustering investigation signifies the structural diversity present in the dataset later utilized for building predictive classification models.

For training and validation of models, this N=225 compound dataset was split with stratified sampling into a training set with n=180 compounds (80%) and an independent hold-out test set with n=45 compounds (20%). Stratification was used to ensure that the relative proportions of inhibitors in Classes A, B, and C were maintained in both subsets. The machine learning model input was a high-dimensional feature vector computed for every compound. After the feature generation step and the initial processing steps in the modeling pipeline (e.g., the elimination of low-variance features), a total of 2268 numerical features were finally used. This rich feature set was intended to capture various aspects of molecular structure and properties, combining 2D physicochemical descriptors, 2048-bit Morgan fingerprints (radius 2), and 3D conformational descriptors, providing a comprehensive representation of each molecule for classification algorithms.

### Performance of the machine learning algorithms

Hyperparameter tuning for the different machine learning algorithms and pipeline setups (Pipeline A: without PCA; Pipeline B: with PCA) was performed using 5-fold stratified cross-validation on (n=180) compound training set, optimizing balanced accuracy. As abstracted in Table 1, ensemble techniques directly applied to the high-dimensional feature space (Pipeline A) exhibited robust performance within this cross-validation process. Random Forest (RF_A) and Extra Trees (ET_A) provided the best mean balanced accuracies of 0.7726 and 0.7690, respectively. Adding PCA (Pipeline B) did not, in general, enhance and sometimes reduced these cross-validation scores (e.g., RF_B: 0.7445; ET_B: 0.7449). Less complex classifiers had moderate (LR_A: 0.7341; KNN_A: 0.7299) to poorer (SVM_A: 0.6961) cross-validation performance.

To ensure that the reported predictive performance was not an artifact of data leakage or model bias, several mitigation strategies were employed. We utilized stratified sampling to partition the training ($n=180$) and test ($n=45$) sets, which preserved the original class distribution across both subsets. Crucially, the independent hold-out test set ($n=45$) was completely sequestered and was not involved in any aspect of model training, oversampling via SMOTE, or hyperparameter tuning. The consistent performance observed between the cross-validation scores and this strictly unseen test partition, achieving a balanced accuracy of 78.46% for the optimal RF_B model demonstrates the model’s true generalization capability and the absence of significant selection bias.

**Table 1:** Summary of hyperparameter optimization results derived from 5-fold stratified cross-validation on the training set (n = 180 compounds). The table lists the best mean balanced accuracy achieved for each tested model configuration during RandomizedSearchCV (40 iterations). Pipeline A involves direct application of the classifier after preprocessing (imputation, low-variance feature removal, scaling, SMOTE), while Pipeline B incorporated Principal Component Analysis (PCA retaining 95% variance) between scaling and SMOTE. Key optimized hyperparameters that yielded the reported best score are shown. Model abbreviations: LR (Logistic Regression), KNN (K-Nearest Neighbors), SVM (Support Vector Machine), RF (Random Forest), ET (Extra Trees), GB (Gradient Boosting).

Model Config	Mean CV Balanced Accuracy	Key Optimized Hyperparameters
RF_A	0.7726	n_estimators=200, min_samples_split=2, min_samples_leaf=1, max_features=’log2’, max_depth=20, bootstrap=True
ET_A	0.7690	n_estimators=600, min_samples_split=5, min_samples_leaf=3, max_features=’log2’, max_depth=None
ET_B	0.7449	n_estimators=200, min_samples_split=10, min_samples_leaf=3, max_features=0.6, max_depth=None
RF_B	0.7445	n_estimators=500, min_samples_split=10, min_samples_leaf=4, max_features=’log2’, max_depth=20, bootstrap=True
LR_A	0.7341	solver=’liblinear’, penalty=’l2’, C=0.05
KNN_A	0.7299	weights=’uniform’, n_neighbors=3, metric=’manhattan’
GB_B	0.7179	subsample=0.7, n_estimators=200, min_samples_split=10, min_samples_leaf=1, max_depth=9, learning_rate=0.2
SVM_A	0.6961	gamma=0.0005, C=100

The generalization performance of these maximally optimized models was subsequently stringently tested on the independent hold-out test set (n=45 compounds) with important performance measures outlined in Table 2. Significantly, the Random Forest model that leveraged PCA (RF_B) performed the best on this novel data with the highest balanced accuracy value of 0.7846 and an overall accuracy of 0.8000. The same model also produced a robust macro F1-score (0.7846).

**Table 2:** Performance metrics evaluated on the independent hold-out test set (n = 45 compounds). The table summarizes the generalization ability of the final optimized models. Metrics shown include overall Accuracy, Balanced Accuracy (robust to class imbalance), and macro-averaged F1-score, Recall, and Precision (giving equal weight to each class). Model abbreviations: LR (Logistic Regression), KNN (K-Nearest Neighbors), SVM (Support Vector Machine), RF (Random Forest), ET (Extra Trees), GB (Gradient Boosting). A/B suffix indicates Pipeline A (no PCA) or Pipeline B (with PCA).

Model Config	Accuracy	Balanced Accuracy	Macro F1-score	Macro Recall	Macro Precision
LR_A	0.6000	0.5692	0.5884	0.5692	0.6157
KNN_A	0.6667	0.6977	0.6665	0.6977	0.6578
SVM_A	0.6889	0.6312	0.6779	0.6312	0.7847
RF_B	0.8000	0.7846	0.7846	0.7846	0.7846
ET_B	0.7556	0.7516	0.7516	0.7516	0.7516
GB_B	0.6444	0.6751	0.6333	0.6751	0.6105
RF_A	0.7778	0.7701	0.7988	0.7701	0.8398
ET_A	0.7778	0.7701	0.7689	0.7701	0.7683

Notably, although RF_A (0.7701 balanced accuracy) and ET_A (0.7701 balanced accuracy) were outstanding while cross-validated as well as strong on the test set, model RF_B was marginally better in generalizing to this unseen test partition. Models such as ET_B (0.7516 balanced accuracy) also did well. The large difference noted here between the high balanced accuracy scores attained when models were refitted on the full SMOTE-sampled training set (results not shown) and the more conservative mean cross-validation and test set scores underscores the value of independent test set assessment to estimate true generalization past perhaps overly optimistic training-phase resampling estimates. The selection of RF_B as the final predictive model, despite its slightly lower mean cross-validation score compared to RF_A, was based on its superior performance on the independent hold-out test set. We prioritized the results from this strictly unseen data as a critical safeguard against potential overfitting to synthetic samples generated during the SMOTE oversampling process in the training phase. Because RF_B demonstrated higher reliability for real-world generalization on this ’gold standard’ independent set achieving the highest balanced accuracy of 78.46%it was chosen as the most robust global model for subsequent virtual screening applications. A fine-grained breakdown of its classification performance is discussed in the following section.

### Detailed performance analysis of the best model (RF_B)

**Fig. 5:**
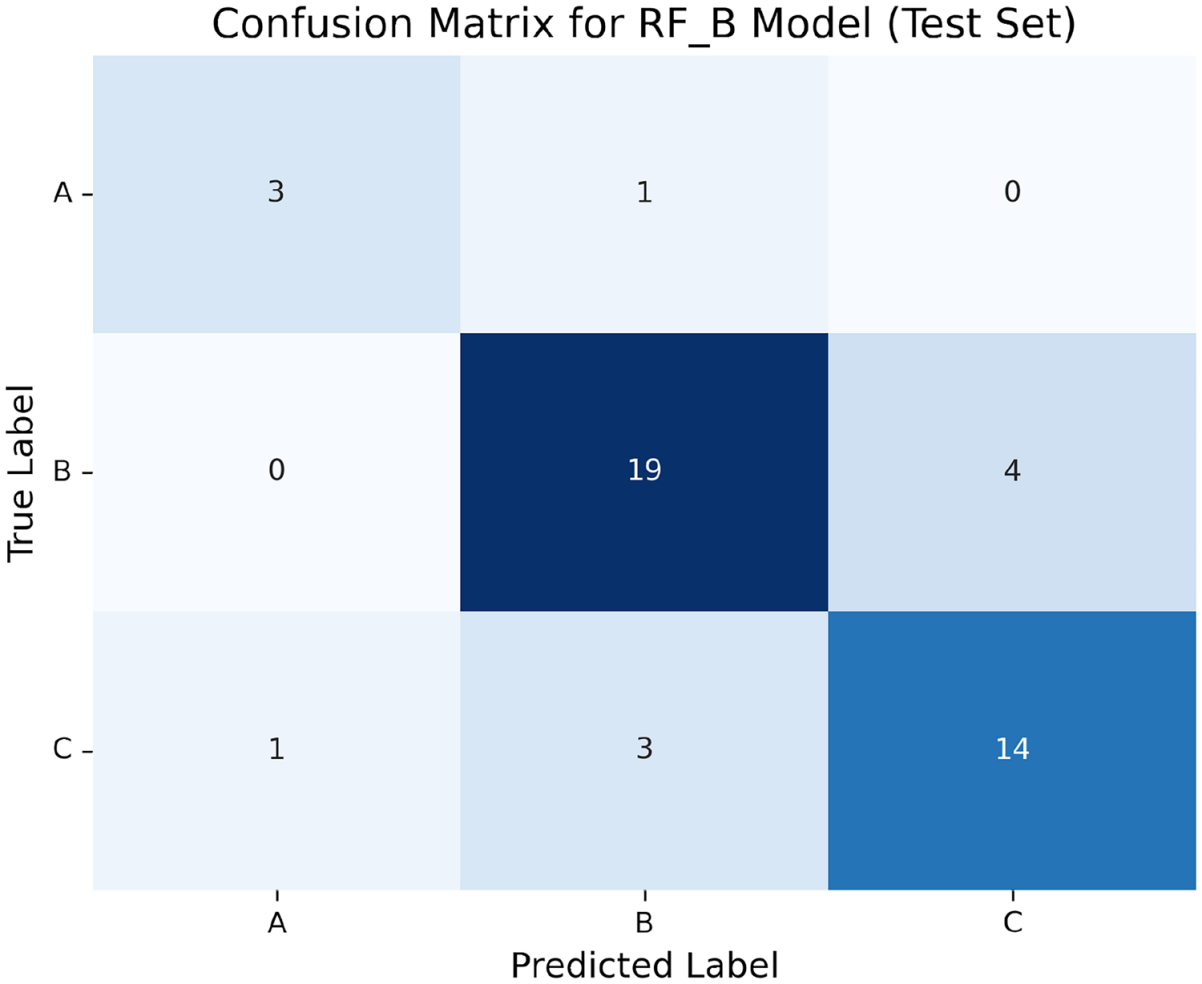
Confusion matrix illustrating the performance of the optimized RF B model on the independent test set (n=45 compounds). Rows correspond to the true activity classes (Top: A, Middle: B, Bottom: C), and columns correspond to the predicted classes (Left: A, Middle: B, Right: C). Cell values indicate the number of compounds in each category. Diagonal elements represent correct classifications. Non diagonal elements represent misclassifications.

To further understand the predictive characteristics of the selected RF B model, its performance on the test set was analyzed in more detail using a confusion matrix and per-class metrics. The RF_B model confusion matrix (Fig. 5) illustrates the classification results. The model accurately predicted 3 of the 4 instances of minority Class A (75% recall). For the majority of Class B, 19 of 23 instances were correctly classified (83% recall). For Class C, 14 of 18 instances were correctly classified (78% recall). The major misclassification was between the neighboring moderate (B) and high (C) activity classes: 4 true Class B compounds were predicted as Class C, and 3 true Class C compounds were predicted as Class B. Moreover, one true Class A compound was predicted as Class B, and one true Class C compound was predicted as Class A. There were no Class A compounds predicted as Class C, or vice versa. The per-class metrics from the classification report (Table 3) offer additional quantitative information. The RF B model exhibited good and balanced performance for the most populated Class B, with both precision and recall at 0.83, yielding an F1-score of 0.83. Performance for Class C was also robust, with precision and recall both at 0.78 (F1-score 0.78). For the challenging minority Class A, the model achieved respectable precision and recall of 0.75 (F1-score 0.75), despite the limited number of test samples for these rarer inhibitors (n=4). The macro-averaged F1-score of 0.78 across all classes further indicates balanced predictive strength across the different activity levels.

**Table 3:** Detailed per-class performance metrics (classification report) for the final selected RF B model evaluated on the independent test set (n=45 compounds). The table shows precision, recall, F1-score, and support (number of true instances) for each activity class (A, B, C). Overall accuracy and macro/weighted averages for precision, recall, and F1 scores across classes are also provided, offering insights into model performance considering potential class imbalance.

Class	Precision	Recall	F1-score	Support (n)
A	0.75	0.75	0.75	4
B	0.83	0.83	0.83	23
C	0.78	0.78	0.78	18
Accuracy			0.80	45
Macro Avg	0.78	0.78	0.78	45
Weighted Avg	0.80	0.80	0.80	45

### Model interpretation using SHAP

**Fig. 6:**
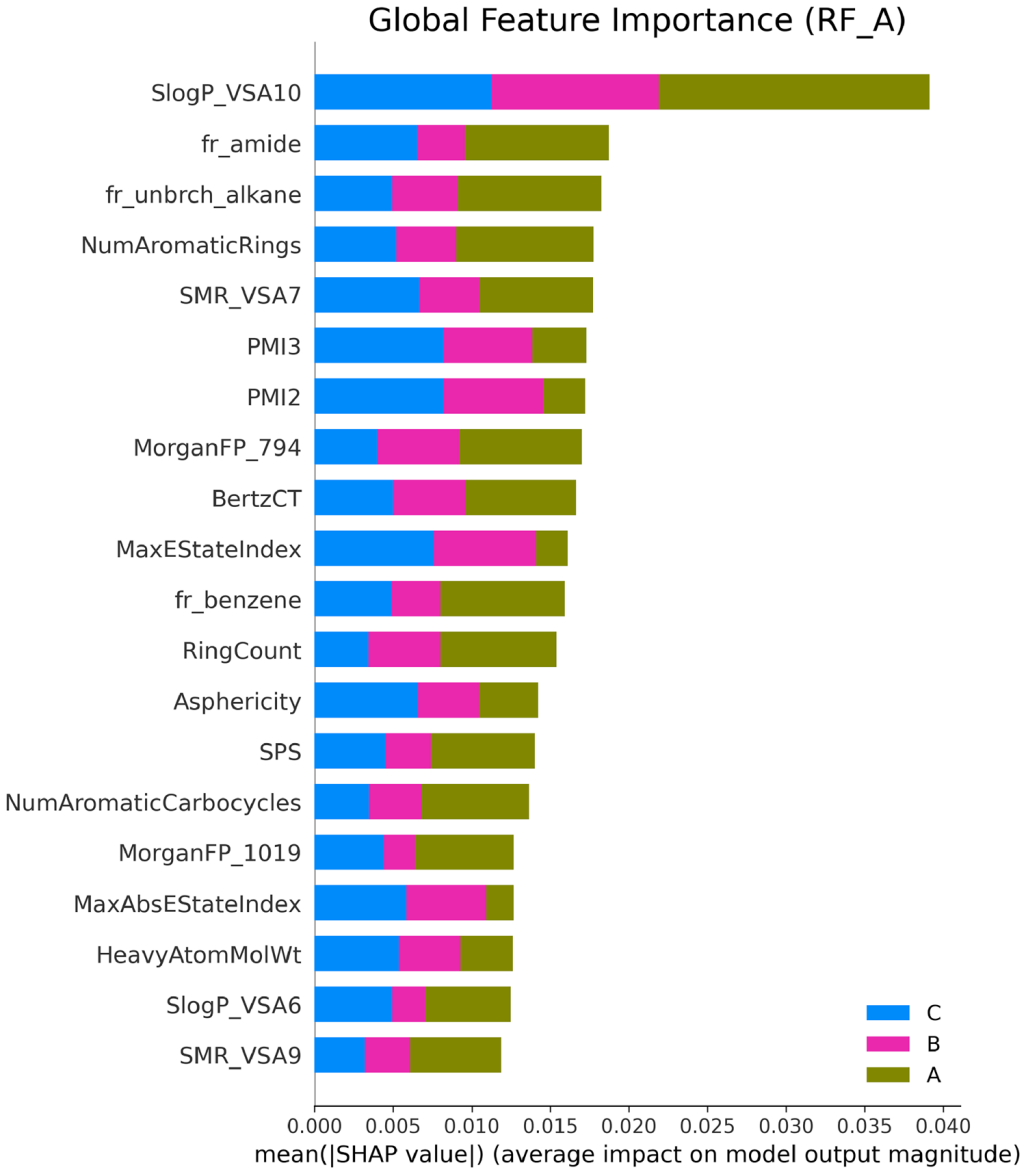
Global feature importance ranking for the optimized RF model (Random Forest), determined by the mean absolute SHAP (SHapley Additive exPlanations) values calculated across all test set instances. Features are ranked from most impactful (top) to least impactful (bottom) based on their average contribution to the model’s output magnitude.

For the sake of explaining the primary drivers of the Random Forest model predictions, a SHapley Additive exPlanations (SHAP) analysis was performed.[^48,53^] This method determines the contribution of each input feature (properly transformed through the pipeline) toward Random Forest’s output on the test set of data. Global feature importance, looking at the mean absolute SHAP value, revealed that the model bases its decision on an entire variety of molecular properties, not just a single dominating factor (see Fig. 6). Such features traversed different chemical domains, including physicochemical properties related to lipophilicity and surface area contributions (SlogP_VSA descriptors), functional group and structural moiety counts (such as counts of amides and aromatic rings), and those relating to molecular shape and complexity (Principal Moments of Inertia, topological indices). Essentially, this means the model does not work on some simplistic criterion but rather conducts a holistic examination of the molecule, combining size, shape, lipophilicity, and the presence of critical pharmacophoric elements into one screening-based classification procedure.

**Fig. 7:**
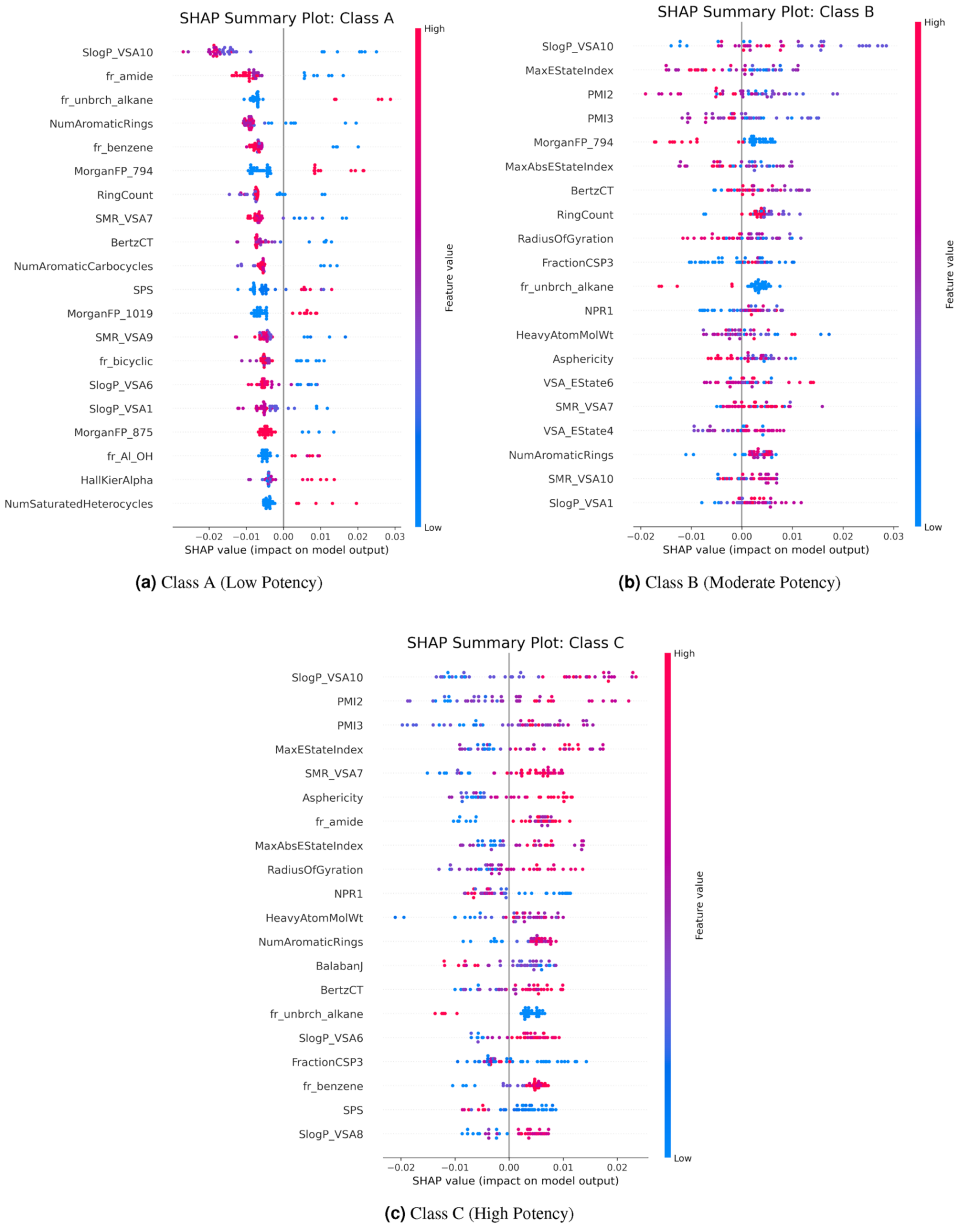
SHAP summary plots illustrating global feature contributions for the RF model predictions across the test set, broken down by predicted activity class. Each point represents a single feature for a single instance. The horizontal position indicates the SHAP value (impact on model output: positive values push prediction towards that class), color indicates the feature’s value (high/low), and vertical position clusters points for each feature. Features are ranked by overall importance based on mean absolute SHAP value.

### Virtual screening

Delving deeper, SHAP summary plots illuminated that the effect of some features varies across the different activity classes, consistent with the interpretation that the model lined up different structure-activity rules for each potency assignment (Fig. 7). Higher descriptor values for molecular size and complexity tended to push predictions toward potency Class C. On the flip side, various Morgan fingerprint bits coding for some specific substructural fragments relatively decreased a Class C prediction, indicating those motifs might somehow be bad for potent inhibition. In contrast, Class A depended differently on features, where descriptors mostly related to simpler structures or decreased lipophilicity moved the predictions toward this lower-potency category. This differential impact speaks to the model learning a more nuanced classification scheme than just a linear scale of ”good” versus ”bad” features. Local force plots can of course be used to further detail these interactions for individual compounds; nonetheless, the global analysis attests to the fact that the Random Forest model learned a well-rounded chemically relevant decision.

To demonstrate the practical application of the optimized RF_B pipeline, the model was evaluated on a curated set of 14 external compounds not present in the original training or testing sets. These structures were subjected to the same rigorous standardization protocol and feature generation pipeline, incorporating 2D, 2048-bit Morgan fingerprints, and 3D descriptors, to ensure full methodological alignment with the model’s training phase. The resulting predictions, along with their associated class probabilities, are summarized in Table 4. The majority of the compounds (11 out of 14) were assigned to the moderate-potency Class B. Notably, the model successfully identified structural diversity within the set, predicting one compound (R5) as high-potency Class C and two compounds (R7 and R8) as low-potency Class A. The identification of Class A candidates highlights the model’s ability to maintain sensitivity for minority structural motifs, even when applied to novel chemical matter.

**Table 4:** Predicted activity classes and associated probabilities for 14 curated external compounds using the validated RF-B model.

Original SMILES	Predicted Class	Prob_A	Prob_B	Prob_C
CCC1=CC(=CC=C1)NC(=O)C2=CC3=C(S2)C4=CC=CC=C4N(C3=O)C	B	0.2624	0.4398	0.2978
CC1=C(C(=O)NC2=C(C=NN12)C(=O)OC)OC3=CC=CC=C3	B	0.3281	0.4126	0.2592
CC1=CC=C(C=C1)C2=C3C=C(C=CC3=NO2)C(=O)NC4=CC=C(C=C4)OC	B	0.2043	0.5488	0.2469
C1=CC(=CC=C1C2=NOC(=N2)C3=C(C=CS3)NC(=O)C4=COC=C4)Cl	B	0.2789	0.3717	0.3494
BrC1=CC=C(C=C1)C2=NOC(=N2)C3=C(C=CS3)NC(=O)C4=COC=C4	C	0.1690	0.3622	0.4688
O=C(C1=CC=NC=C1)N/N=C/C1=CN(CC2=CC=CC=C(C#N)C2)C3=C1C4=CC=CC=C4N3	B	0.2484	0.4283	0.3233
O=C(C1=CC=NC=C1)N/N=C/C1=CN(CC2=CC=CC=C([N+]([O-])=O)C2)C3=C1C4=CC=CC=C4N3	A	0.3669	0.3147	0.3184
O=C(C1=CC=NC=C1)N/N=C/C1=CN(CC2=CC=CC=C(Cl)C2)C3=C1C4=CC=CC=C4N3	A	0.3435	0.3396	0.3169
O=C(C1=CC=NC=C1)N/N=C/C1=CN(CC2=CC(F)=CC=C2)C3=C1C4=CC=CC=C4N3	B	0.2621	0.4070	0.3309
O=C(C1=CC=NC=C1)N/N=C/C1=CN(CC2=CC=CC=C(C(F)(F)F)C2)C3=C1C4=CC=CC=C4N3	B	0.2413	0.4275	0.3312
O=C(C1=CC=NC=C1)N/N=C/C1=CN(CC2=CC(F)=C(C(F)(F)F)C=C2)C3=C1C4=CC=CC=C4N3	B	0.1698	0.4769	0.3533
O=C(C1=CC=NC=C1)N/N=C/C1=CN(CC2=CC=C=C2F)C3=C1C4=CC=CC=C4N3	B	0.2567	0.4208	0.3226
O=C(C1=CC=NC=C1)N/N=C/C1=CN(CC2=CC=C(C(F)(F)F)C=C2)C3=C1C4=CC=CC=C4N3	B	0.1648	0.4955	0.3397
CC2=CC=C1OC(=NC1=C2)C3=CC=C(C=C3)NC(=O)NC4=CC=C(C=C4)C6=NC5=C(C=CC(=C5)C)O6	B	0.1322	0.4458	0.4220

**Fig. 8:**
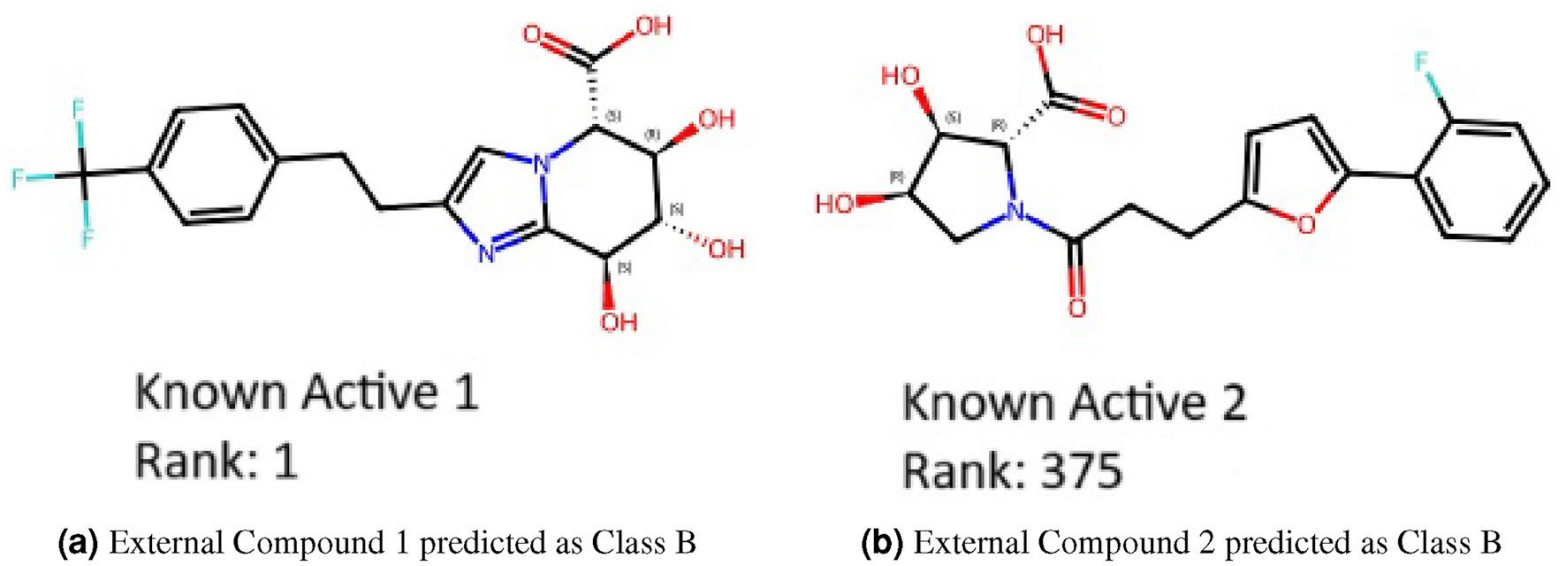
Predictions by the RF_B model for two representative external compounds. These examples illustrate the model’s application to novel chemical entities not present in the training data. Rank refers to predicted probability-based prioritization.

There are several structural motifs and functional groups that seem to have been identified by the algorithm. Most predicted compounds contain a common hydrazone-based core (>C=N–NH–) of a tricyclic carbazole-like type, suggesting the model’s capability to recognize and generalize in a particular series of compounds. This series’ diversity results from multiple substitutions on a terminal benzyl group, including cyano (–C$\equiv$N), nitro (–NO$_2$), halogen substitutions (Cl, F), and electron-withdrawing trifluoromethyl (–CF$_3$) groups. Other than this primary series, the model also identified other unique chemotypes, such as a thieno[2,3-b]indole derivative, a pyrazolone, an isoxazole, and a complex large bis-urea linked scaffold, showing its versatility across multiple structural families. All 14 curated compounds were assigned to an activity class with varying levels of confidence. The model identified one high-potency candidate (R5) as Class C and two low-potency candidates (R7 and R8) as Class A, with the remainder categorized as Class B. While the absolute probability scores (ranging from 0.34 to 0.59) are more conservative than previous iterations, they represent a more reliable assessment derived from the integrated $2\text {D}+3\text {D}$ feature space. For instance, the identification of the dichlorinated carbazole derivatives (R7 and R8) as Class A demonstrates the model’s sensitivity to structural motifs that may have previously been over-predicted as moderate hits. These 14 compounds have not been previously reported as HPSE inhibitors in the available literature or public databases. Those predicted as Class C, or those exhibiting consistent Class B profiles across both $2\text {D}$ and $3\text {D}$ feature sets, are identified as primary potential hit compounds for experimental validation (Fig. 8).

## Discussion

This research designed and validated a machine learning pipeline capable of multi-class inhibition classification for human Heparanase (HPSE), with the Random Forest combined with PCA (RF_B) deemed the best model. The latter demonstrated good general applicability with an independent test set, showing around 78.5% balanced accuracy and 80% overall accuracy. These predictive capabilities highlighted by the equally strong F1 score (0.785) and ROC AUC (0.877) denote the ability to largely capture complex structure-activity relationships, which govern HPSE inhibition. The model chose to support the minority Class A (reinforced by a 75% recall) using SMOTE for training, most of whose misclassifications came in the form of states in-between Class B and C potency ranges. However, while ensemble methods performed well in general^12^, the addition of PCA into RF_B enhanced the generalization capacity of testing over its non-PCA equivalent (RF_A), even though RF_A had obtained slightly better cross-validation scores. This finding underscores the critical importance of independent test set validation, as cross-validation results, particularly when combined with resampling techniques like SMOTE, may not fully reflect real-world generalization potential.

**Fig. 9:**
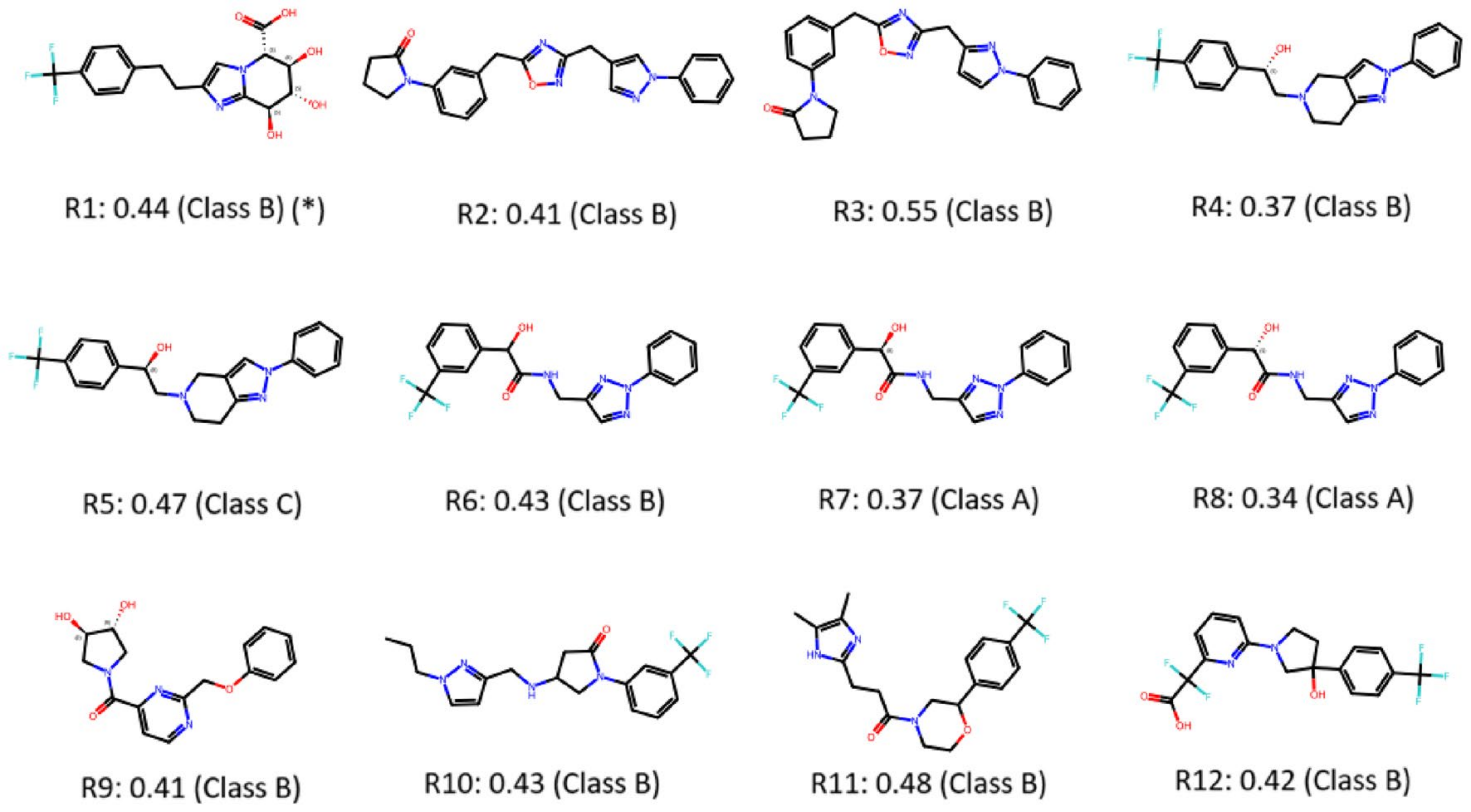
Examples of activity class predictions by the RF B model for a selection of 12 diverse external compounds (labeled R1-R12). Each entry displays the 2D chemical structure, its identifier (R1-R12), the model prediction score, and the predicted class (e.g., P-B for predicted Class B, P-A for predicted Class A).

Robustness of the model is attributable to methodological rigor, especially stringent data curation and the use of a diverse feature range spanning 2D, fingerprint, and 3D descriptors. Regarding the model’s reliability, the Applicability Domain (AD) was defined operationally through our stringent curation pipeline. While a statistical distance-based AD (e.g., leverage or Euclidean distance) was not explicitly calculated, we ensured that all compounds in the test and virtual screening sets adhered to the same physicochemical boundaries as the training set, such as a Molecular Weight (MW) $\le 500$ Da. Furthermore, structural relevance was maintained by confirming that key molecular scaffolds, such as the tricyclic carbazole-like types identified in our external set, were well-represented within the training distribution. These boundaries define the chemical space where our model’s predictions remain most reliable. The PCA step within the optimum model procedure, which lends complexity to direct interpretation, was counterbalanced by SHAP analysis of a very similar random forest model, which confirmed that a diverse set of features contributes vastly to classification. Descriptors weighted for lipophilicity-surface area (such as SlogP_VSA10), presence or absence of certain fragments (like fr_amide), 3D shape descriptors (like PMI2, PMI3), and substructural fingerprints (such as MorganFP_794) were all ranked high in importance. Hence, this means the model used a mixture of physicochemical, structural, and shape information as opposed to relying on a single feature type. A key design consideration in this study was the selection of a multi-class classification framework over traditional regression modeling.[^59^] While regression utilizes the full fidelity of continuous $pIC_{50}$ data, classification was prioritized to serve as a robust ’triage’ tool for virtual screening. In early-stage drug discovery, binning compounds into actionable priority tiers, such as the High Potency Class C, is often more practical than predicting precise affinity values, which can be significantly affected by experimental noise and inter-laboratory variability inherent in aggregated public datasets like ChEMBL. Despite the challenges posed by class imbalance, particularly for the minority Class A ($n=18$), the use of SMOTE within an automated pipeline allowed the model to maintain sensitivity across distinct potency levels. This categorical approach provides a more reliable foundation for hit prioritization and guidance during initial screening campaigns than regression models attempting to fit precise but potentially noisy bioactivity values. From a practical standpoint, the validated workflow serves as an efficient filter for heparanase (HPSE) drug discovery during large-scale virtual screening and hit prioritization. The robust performance on external compounds, specifically the identification of distinct potency tiers for the carbazole-hydrazone series (e.g., R7 and R8 as Class A and R5 as Class C) as shown in Fig. 9), supports the utility of integrating 3D shape and electrostatic descriptors with 2D structural information for reliable bioactivity prediction.

Despite the promising predictive performance of the RF_B model, several limitations to this study must be acknowledged. These include the limitations associated with the use of public domain data from the ChEMBL database, the relatively small size of the dataset (N = 225), especially for the minority class of Class A, and the loss of detail in the approach, which was based on classification instead of regression. A key limitation of the current framework is that it does not explicitly model inactive compounds (pIC50 < 4), as these were excluded during dataset curation. While this enables clearer class boundaries, it limits the applicability of the model as a universal screening tool where inactive compounds are commonly encountered. In addition, several methodological decisions also impose limitations on the study. These include the limitations in Morgan fingerprints, where the method, while efficient for the identification of local topological features, does not include three-dimensional spatial relationships and is susceptible to bit collisions. Similarly, the constraints in using the 3D descriptors, where only single conformation, energy-minimized structures were considered, failed to account for flexibility or binding-induced conformational changes. Furthermore, structure-based methods such as docking could provide more realistic ligand conformations within the HPSE binding pocket compared to gas-phase optimized structures. The use of SMOTE also imposes disadvantages, where synthetic data, while efficient for increasing the number of samples, does not reflect the overall chemical space. Finally, the predicted novel compounds were not further validated using structure-based approaches. Future studies must address this by incorporating in silico methods, such as molecular docking or binding affinity estimation, before proceeding to rigorous experimental validation, alongside expanding the dataset with diverse chemical matter and exploring advanced architectures like graph neural networks to fully evaluate this workflow’s applicability for discovering novel HPSE inhibitors.[^39,61,62,63^]

## Conclusion

The approach describes the successful development and validation of a robust machine learning pipeline for multi-class classification of Human Heparanase (HPSE) inhibitors. The best model, RF_B (Random Forest with Principal Component Analysis), showed exceptional generalization ability on an independent test set, reaching 80.00% overall accuracy and 78.46% balanced accuracy. Therefore, it can be stated that the present model is capable of capturing the complex structure-activity relationship governing HPSE inhibition in three potency classes: A, B, and C. This was realized by including several molecular representations in the models, such as 2D, fingerprint, and 3D descriptors, thus taking into account a holistic view of their properties. To this end, the validated RF_B model is an effective, efficient filter in virtual screening campaigns and hit prioritization processes in drug discovery for HPSE, as demonstrated in this work by predicting, with high confidence, a dataset of unseen compounds. Future work should prioritize moving beyond the established ensemble methods. This includes investigating Graph Neural Networks (GNNs), which naturally process molecular structures and can better capture complex features. Furthermore, developing QSAR regression models alongside the current classification model is essential to predict continuous $pIC_{50}$ values, allowing for finer, more quantitative guidance during the lead optimization phase of drug discovery.

## Data Availability

Original data generated and analyzed during this study are included in the CHEMBL Database, HPSE targets and Binding DB[^60^ which is also listed in References.
